# Supplementary material for: Density functional theory studies of MTSL nitroxide side chain conformations attached to an activation loop
Source: Theor Chem Acc. 2016 Mar 23;135:97. doi: 10.1007/s00214-016-1859-z (PMC4805727; doi:10.1007/s00214-016-1859-z)
Supplement: Supplementary file 1 — Supplementary material 1 (DOCX 183 kb) [file 214_2016_1859_MOESM1_ESM.docx]

**Electronic supporting information (ESI):**

**Calculation of EPR parameters (*g*- and *A*-tensors)**

The EPR parameters were calculated using the Gauge-Independent Atomic Orbital (GIAO)^1^ method, the B3LYP hybrid functional and the polarized spilt-valence N07D basis sets.^2-3^ These basis set was optimized for second- and third-row atoms and has already been used with success for accurate calculation of the magnetic tensors (*△g_ii_* = ±0.0005, *△A_ii_* = ±1 G) in gas phase and in solution of nitroxide radicals at a reasonable computational cost and can be downloaded from the DREAMSLAB website.^3^ The Polarizable Continuum Model (PCM) was used to describe solvation in water since the experimental EPR spectrum was measured in water.^5^ All the EPR spectra were simulated using the open source Spinach software library^6^, the Euler angles between frames were determined using Spinach GUI.^7^  The static powder simulation of the W-band spectrum at 150 K was performed using the powder context with the Levedev spherical grid rank 131. All the simulations were performed using one component and no line broadening parameters were included.

**Experimental section**

The expression construct for Aurora-A 122-403 T287A, T288C, C290A, C393A was produced in earlier work for recombinant expression of the protein in E. coli with a N-terminal TEV cleavable His6-tag.^8^ Expression and purification of the kinase was carried out as stated in Burgess & Bayliss (2015) with the following modifications.^9^ After TEV cleavage and affinity chromatography to remove the His6-tag and His6-tagged TEV, 5 mM DTT was added to Aurora-A containing fractions and left to incubate overnight at 4 °C to ensure C288 was in the reduced form to allow subsequent modification with MTSL. The reducing agent was removed by desalting the protein on a HiPrep 26/10 Desalting column as per the manufacturer’s instructions (GE Healthcare) into 20 mM Tris pH 7.0, 0.2 M NaCl, 5 mM MgCl2 & 10 % (v/v) glycerol (EPR buffer). 10-fold excess MTSL was added to the kinase and incubated overnight at 4 °C in order to spin label the kinase. Any remaining impurities, aggregated protein and excess MTSL were removed by size-exclusion chromatography using a HiLoad 16/600 Superdex 200 pg column as per the manufacturer’s instructions (GE Healthcare) into EPR buffer. SDS-PAGE analysis was used to identify fractions of high purity MTSL-Aurora-A 122-403 T287A, T288C, C290A, C393A which were concentrated and flash-frozen for future experiments. 50 μM MTSL-Aurora-A 122-403 T287A, T288C, C290A, C393A was used for CW EPR studies. The spin labelling efficiency was equal to 86% as measured following a published procedure.^10^

93.778 GHz CW measurements were performed on a Bruker E560 spectrometer. The magnetic field was calibrated using a Mn2+ power standard (0.02% MgO) and the procedure described by O. Burghaus et al.^11^ The rigid limits experimental spectrum was recorded at 150 K. Dual-scan measurements were made in order to avoid hysteresis effects and a modulation frequency of 100 KHz and low microwave power (0.004800 mW) were used to avoid distortion of the lineshape.

**Table S1:** Torsional angles of conformations (C1 to C6) of MSTL and corresponding calculated *g*-tensor. All the simulations were performed considering a molecular reference frame that has the origin in the N atom of the nitroxide with the *z* axis parallel to the N-*p_z_* orbital and the *x* axis parallel to the NO bond, the *g*- and *A*-tensors collinear with it. *α*, *β*, *γ* are the Euler angles between the molecular frame and another frame that has origin in the C_α_ carbon with the *z* axis perpendicular to the plane N-C_α_-CO and the *x* axis parallel to the C_α_-C_b_ bond. Comparable values of the hyperfine *A-*tensor were obtained for the 6 conformations that are equal to *A_xx_* = 8 Gauss, *A_yy_* = 7 Gauss and *A_zz_* = 34 Gauss.

|  | $\chi_{1}$*(°)* | $\chi_{2}$*(°)* | $\chi_{3}$*(°)* | $\chi_{4}$*(°)* | $\chi_{5}$*(°)* | *α* | *β* | *γ* | $g_{xx}$ | $g_{yy}$ | $g_{zz}$ |
| --- | --- | --- | --- | --- | --- | --- | --- | --- | --- | --- | --- |
| C1 | -160 | 180 | -90 | 180 | -90 | 0 | 0 | 0 | 85 | 62 | 20 |
| C2 | -160 | 180 | -90 | 180 | +90 | -60 | 0 | 0 | 86 | 61 | 20 |
| C3 | -160 | 180 | -90 | -70 | +90 | +20 | 0 | 0 | 85 | 61 | 19 |
| C4 | -160 | 180 | +90 | 180 | -90 | +50 | +150 | +120 | 86 | 61 | 20 |
| C5 | -160 | +80 | -90 | 180 | -90 | +35 | +80 | -30 | 86 | 60 | 19 |
| C6 | -60 | 180 | -90 | 180 | -90 | -30 | +260 | +10 | 86 | 62 | 19 |

Fig. S1 shows the comparison between experimental and simulated 94 GHz EPR spectra calculated using parameters magnetic parameters shown in Table S1.

**Fig. S1:** Comparison between experimental and theoretical 94 GHz EPR spectra calculated using conformations C1–C6 described in Table S1. The experimental spectrum was measured at 150 K.

Simulated EPR spectra were seen to reproduce well the experimental powder 94 GHz EPR spectrum of the MTSL spin-labelled Aurora-A kinase, the frequency of measurement and experimental conditions were chosen since they provide high resolution of *x*, *y* and *z* principal components of the *g*- and hyperfine *A*-tensors.^12^ This indicated that the system can be described as an isotropic distribution of conformations with different geometry, and the orientation of *A*- and *g*-tensors with respect to specific reference frame was considered lost.

**References**

1. Cheeseman J. R., Trucks G. W., Keith T. A., Frisch M. J. (1996) *J. Chem. Phys.* 104:5497-5509.
2. Barone V., Bloino J., Biczysko M. (2010) *Phys. Chem. Chem. Phys.* 12:1092-1101.
3. Barone V., Cimino P., Stendardo E. (2008) *J. Chem. Theory Comput.* 4:751 –764.
4. Double and triple-ζ basis sets of N07 family, are available to download, available http://dreamslab.sns.it/?pag=downloads, accessed on October, 2015.
5. Tomasi J., Mennucci B., Cammi R. (2005) *Chem. Rev.* 105:2999-3093.
6. Hogben H. J., Krzystyniak M., Charnock G.T.P., Hore P. J., Kuprov I. (2011) *J. Magn. Reson.* 208:179 –194.
7. Biternas A., Charnock, G.T.P. Kuprov, I. (2014) *J. Magn. Reson.* 240:124-131.
8. Rowan F. C., Richards M., Bibby R. A., Thompson A., Bayliss R., Blagg J. (2013) *ACS Chem. Biol.* 8:2184 –2191.
9. Burgess S. G., Bayliss R. (2015) *Acta Crystallogr. F. Struct. Biol. Commun.* **71**, 315.
10. Persson M., Harbridge J. R., Hammarström P., Mitri R., Mårtensson L., Carlsson U., Eaton G. R., Eaton S. S. (2001) *Biophys. J.* 80:2886 –2897.
11. Burghaus O., Rohrer M., Gotzinger T., Plato M., Mobius K. (1992) *Meas. Sci. Technol.* 3:765-774.
12. Nesmelov Y. E., Thomas D. D. (2010) *Biophys Rev.* 2:91-99.
